# Cyclic intensive light exposure induces retinal lesions similar to age-related macular degeneration in APPswe/PS1 bigenic mice

**DOI:** 10.1186/1471-2202-13-34

**Published:** 2012-03-24

**Authors:** Zhizhang Dong, Juan Li, Yunxia Leng, Xuerong Sun, Huiling Hu, Yuan He, Zhiqun Tan, Jian Ge

**Affiliations:** 1 State Key Laboratory of Ophthalmology, Zhongshan Ophthalmic Center, Sun Yat-sen University, Guangzhou, China; 2 Department of Neurology, University of California Irvine School of Medicine, Irvine, CA 92697, USA; 3 Institute for Memory Impairments and Neurological Disorders, University of California Irvine School of Medicine, Irvine, CA 92697, USA

## Abstract

**Background:**

Intensive light exposure and beta-amyloid (Aβ) aggregates have been known as a risk factor for macular degeneration and an important component in the pathologic drusen structure involved in this disorder, respectively. However, it is unknown whether Aβ deposition mediates or exacerbates light exposure-induced pathogenesis of macular degeneration. Several studies including the one from us already showed accumulation of Aβ deposits in the retina in Alzheimer's transgenic mice. Using histopathological analysis combined with electroretinographic functional assessment, we investigated the effects of cyclic intensive light exposure (CILE) on the architecture of retina and related function in the APPswe/PS1bigenic mouse.

**Results:**

Histopathological analysis has found significant loss of outer nuclear layer/photoreceptor outer segment and outer plexiform layer along with abnormal hypo- and hyper-pigmentation in the retinal pigment epithelium (RPE), remarkable choroidal neovascularization (CNV), and exaggerated neuroinflammatory responses in the outer retina of APPswe/PS1 bigenic mice following cyclic intensive light exposure (CILE), whereas controls remained little change contrasted with age-matched non-transgenic littermates. CILE-induced degenerative changes in RPE are further confirmed by transmission electron microcopy and manifest as formation of basal laminar deposits, irregular thickening of Bruch's membrane (BrM), deposition of outer collagenous layer (OCL) in the subretinal space, and vacuolation in the RPE. Immunofluorescence microscopy reveals drusenoid Aβ deposits in RPE as well as neovessels attached which are associated with disruption of RPE integrity and provoked neuroinflammatory response as indicated by markedly increased retinal infiltration of microglia. Moreover, both immunohistochemistry and Western blots detect an induction of vascular endothelial growth factor (VEGF) in RPE, which corroborates increased CNV in the outer retina in the bigenic mice challenged by CILE.

**Conclusions:**

Our findings demonstrate that degenerative changes in the outer retina in the APPswe/PS1 bigenic mouse induced by CILE are consistent with these in AMD. These results suggest that an Alzheimer's transgenic animal model with accumulation of Aβ deposits might be an alternative animal model for AMD, if combined with other confounding factors such as intensive light exposure for AMD.

## Background

Age-related macular degeneration (AMD) is a degenerative disease in the eye, which causes irreversible blindness in elderly and is one of the major causes of blindness in developed countries [1]. Drusen and choroidal neovascularization (CNV) are the two pathological hallmarks of AMD, of which drusen accumulates in the subretinal pigment epithelium (RPE) space and CNV is characterized by new angiogensis from choroidal blood vessels which break through Bruch's membrane (BrM) and RPE layer and is often associated with subretinal hemorrhage [2]. Recent studies suggest that beta-amyloid (Aβ) peptide, a major molecular signature in the brain of Alzheimer's disease, might play an important role in the pathogenesis of AMD [3]. Aβ aggregates have been identified as one of the major components in drusen as well as in RPE cells in the retina of AMD [4-7]. Similarly to the brain, several groups of investigators including us also demonstrate perivascular deposition of Aβ in the retina in human CNV as well as different lines of Alzheimer's-related transgenic mice [8,9]. Importantly, immunotherapy that targets Aβ significantly attenuated retinal lesions and improved retinal function in an AMD mouse model [10,11]. Moreover, growing evidence has indicated smoking [12], extensive sun light exposure [13], and ageing [14] as important risk factors for AMD. CILE is detrimental to the BrM, RPE, photoreceptor and other retinal structures due to induction of the reactive oxygen species and inflammatory response [15,16]. CILE induced drusen formation or stimulated CNV through upregulation of vascular endothelial growth factor (VEGF) as well as induction of oxidative stress in rodent models [17-20].

Nevertheless, the molecular basis of the pathogenesis of AMD, particularly about the role of Aβ deposition in the development of RPE lesions and CNV, remains elusive. In this study we examined the effects of constitutional expression of Aβ deposits on retinal lesions induced by CILE in the APPswe/PS1 bigenic mouse model of Alzheimer's disease, and found that CILE significantly increased Aβ deposition linked with AMD-like retinopathies in the transgenic mice. By contrast, there were no significant changes in the retina of either non-transgenic mice received equal light exposure or age-matched transgenic control.

## Results

### Cyclic intensive light exposure induces abnormal pigment deposition in RPE, CNV and degenerative changes in the retina of APPswe/PS1 bigenic mice

**Figure 1:**
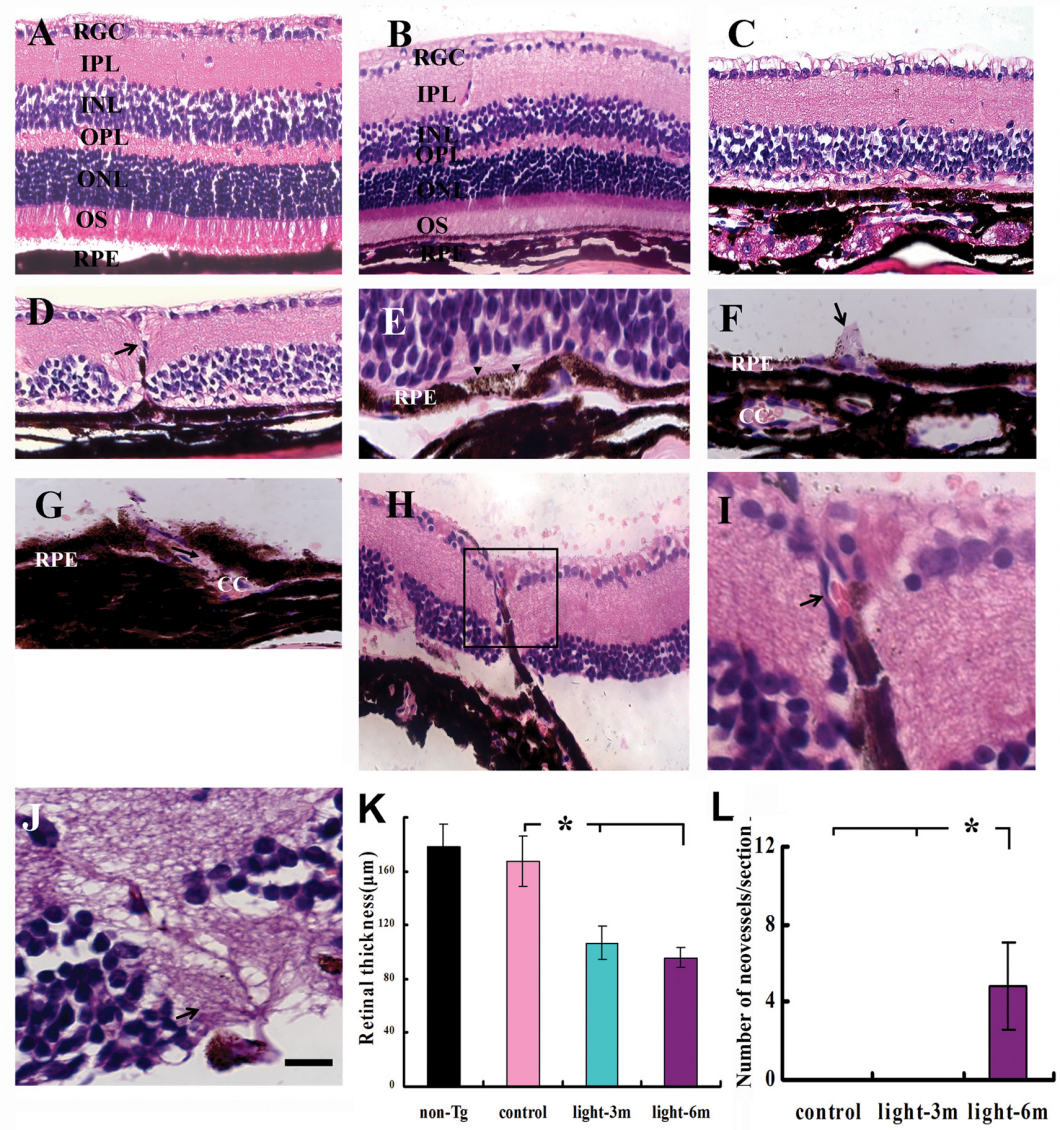
**Degenerative changes and neovascularization in the retina of bigenic mice following cyclic intensive light exposure**. Hematoxylin and eosin (H & E) staining was performed on retinal cross sections as described in Methods. (**A**) A part of a retinal cross section through the optic nerve head of a 12-month old non-Tg control mouse (non-Tg) showing normal architecture and morphology. (**B**) A part of a retinal cross section from an age-matched APPswe/PS1 bigenic control mouse (control) (demonstrates similar morphology to the non-Tg control). (**C**) A part of a retinal cross section from a bigenic mouse following 3-month CILE (light-3 m) shows remarkable thinning outer nuclear layer/photoreceptor outer segment. (**D**) A part of a retinal cross section from a bigenic mouse following 6-month CILE (light-6 m) exhibits thinning neuroepithelial layers and newly-developed vessels. (**E-G**) Cross sections from the bigenic mice after 6-month CILE showing hypo-pigmentation (E, arrowheads), proliferating RPE (F, arrow), hyperpigmentation and a newly-developed vessel originated from choroicapillaris disrupt the RPE layer with red blood cells inside (G, arrow) in the RPE layer. (**H-J**) Show different types of newly-developed vessels that disrupt retinal architecture. Boxed area in (H) is shown in (I) at a higher magnification, in which the arrow indicates a red blood cells in the newly-developed vessel. (**K**) Quantification of the retinal thickness for each group of animals. (**L**) Quantification of neovessels in the outer retina on the cross section for each group of animals. Bars depict mean ± SEM. *: P < 0.001 (*N *= 6-8). CC: choroicapillaris; RPE, retinal pigment epithelium; OS, outer segment; ONL, outer nuclear layer; OPL, outer plexiform layer; INL, inner nuclear layer; IPL, inner plexiform layer; RGC, retinal ganglion cell layer. Scale bars = 50 μm for (A-D), 20 μm for E, F, G, J, 50 μm for H, 15 μm for I.

To evaluate the effect of CILE on the retina of mice, the fundus was examined before and after CILE based on fundus photographs. Apparently, increased pigment deposits and shrunken vessels were detected in APPswe/PS bigenic mice after CILE, particularly in these after 6-month CILE compared with age-matched control or non-Tg mice after the exposure (Additional file 1, Figure 6). Nevertheless, neither yellowish retinal deposits/drusen nor retinal hemorrhage was found in the fundus photos from both bigenic and non-Tg mice. These observations are in agreement with conventional light microscopic analysis following H&E staining on retinal cross sections (Figure 1). There is no conspicuous difference in the architecture of the retina between a non-Tg (Figure 1A) and an age-matched bigenic control (Figure 1B) mouse. By contrast, a series of remarkable degenerative changes are visible in the retina in all the animals from the groups of bigenic mice following CILE (Figures 1C-J) compared with the control (Figure 1B). Significant loss of the outer nuclear layer (ONL)/photoreceptors is visible following 3-month CILE (Figure 1C), the entire outer plexiform layer (OPL) and ONL/photoreceptor outer segment (OS) layer are barely remaining after 6-month CILE (Figures 1D, H, and 1I), which is consistent with the thickness loss as confirmed by thickness measurement (Figure 1K). Importantly, abnormal pigment deposition, i.e., hypopigmentation (Figure 1E) or hyperpigmentation which appears in association with RPE proliferation as indicated by the hypertrophic appearance of RPE cells in the retina in the bigenic mice after 6-month CILE (Figure 1F). Moreover, CILE-induced CNV is usually shown here as the disruption of the Bruch's membrane (BrM) due to invasion of new vessels that enclose red blood cells from choroicapillaris (CC) (Figures 1D, G-J). Quantification of newly generated vessels shows 75% of the mice (9 of 12) with about 4.8 ± 2.2 evident new vessels per cross retinal section in the group with 6-month CILE, but no new vessel was detected in the other groups at all (Figure 1L). In addition, no typical drusen structures were found in these mice.

**Figure 2:**
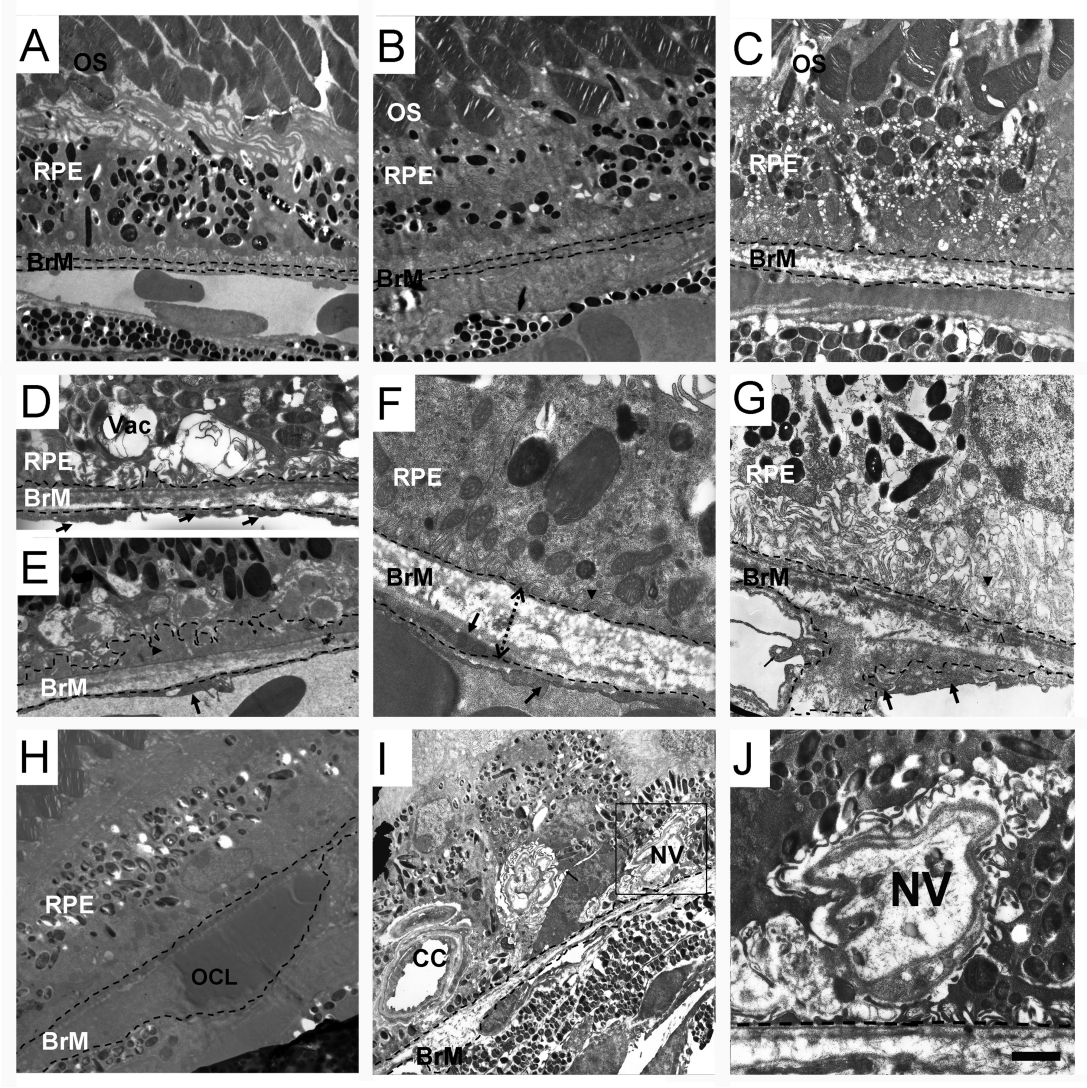
**Transmission electron microscopic analysis of retina**. Transmission electron microscopy was conducted as described in the Methods. Representative micrographs covering the RPE regions are shown here. (**A**) Normal morphology of photoreceptor outer segment (OS), RPE and Bruch's membrane (BrM) is detected in non-Tg control mouse. (**B**). APP/PS1 bigenic control mouse retina demonstrates slight degenerating changes in the RPE but with normal morphology of Bruch's membrane. (**C-J**) In the retina from the group of bigenic mice following 6-month CILE (light-6 m), micrographs show massive vacuoles in the RPE cells and disrupted photoreceptor outer segments (C), vacuoles in RPE cells at a higher magnification (D), degenerative changes with small basal laminar deposits in RPE (E, arrowhead, dashed line enclosure), abnormal appearance of basal infoldings (G, arrowhead), breaks of the elastic layer of Bruch's membrane (G, open arrowheads), and thickened or distorted Bruch's membrane (BrM) in RPE cells (F&G). Bold arrows in (D-J) indicate endothelial fenestrations of choriocapillaris (CC) and infiltration of microglia (G&I, thin arrows) is seen in the RPE-Bruch's membrane interface. (H) The outer collagenous layer (OCL) is observed adjacent to Bruch's membrane. (I) Neovascularization (NV) and adjacent capillary (CC) are found on both sides of Bruch's membrane. An image from an adjacent section to the boxed area in (I) is shown in (J) at a higher magnification. Scale bar = 4 μm forA, B, H, I, 2 μm for E, 1 μm for, 3 μm for C, G, 1.5 μm for D, F, and J.

To evaluate the CILE-induced pathological lesions in the retina further, we performed transmission electron microscopy to examine the ultrastructure in the retina. A representative micrograph demonstrates typical normal ultrastructure of the outer retina in the 12-month old non-Tg mice (Figure 2A). There is no obvious difference in the general ultrastructure as well as the RPE-BrM-CC complex between non-Tg and age-matched APPswe/PS1 bigenic controls (Figure 2B). Conversely, intracellular vacuoles along with membranous debris (Figures 2C, D), small basal laminar deposits (Figure 2E), and thickened BrM (Figures 2C, F) are commonly seen in the RPE layer in the group of bigenic mice that received 6-month CILE. Noticeably, shrunken and disrupted architecture of basal infoldings of RPE is also detected in most eyes from these animals as well (Figures 2F, G, and 2J). In some cases, loss of the continuity of BrM elastic layer (Figure 2G), outer collagenous layer deposition (Figure 2H), and infiltration of microglia (Figures 2G and 2I) around the BrM-CC complex are evident as previously demonstrated [21,22]. Importantly, CILE also induces invasion of choroicapillaris into the RPE layer resulting in formation of neovessels, which usually demonstrate as vascular buds as shown in Figures 2I and 2J.

### Cyclic intensive light exposure-induced retinal lesions are associated with accumulation of β-amyloid deposits in the RPE of APPswe/PS1 bigenic mice

**Figure 3:**
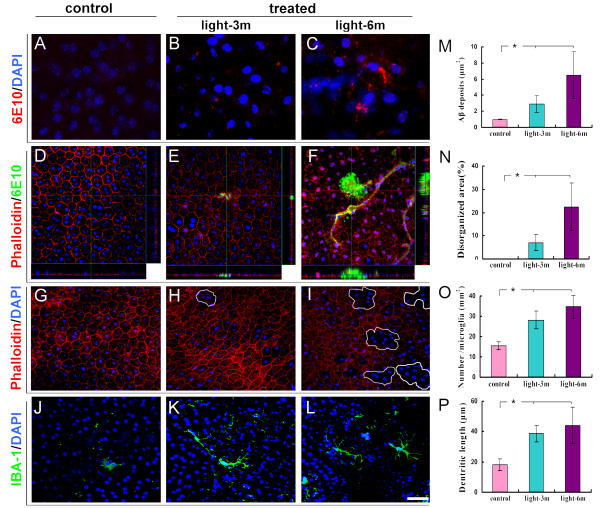
**Immunofluorescence microscopy of RPE flatmouts**. (**A**-**C**) Dissected RPE flatmounts from a control and treated with CILE of APPsw/PS1 bigenic mice were stained with 6E10 IgG for Aβ (red). (**D-F**) RPE flatmounts were double-stained with 6E10 IgG for Aβ (green) and rhodamine-conjugated phalloidin for F-actin (red) and visualized by confocal microscopy. Druen-like Aβ deposits and vessel-like structures are evident in the mice following 6-month CILE (F). (**G**-**H**) Fluorescent microscopy reveals normal morphology of RPE cells by phalloidin staining (red) in the control (G) and increased defects of RPE morphology (white circles) in mice received CILEs (H & I). (**J**-**L**) Fluorescent immunoreactivity of IBA-1, a molecular marker for microglia, shows markedly increased number of microglia and microglial dendritic staining (green) in RPE from the mice following CILEs (K & L) compared with the control (J). Cell nuclei are counterstained blue by 4'-6-diamidino-2-phenylindole (DAPI). Scale bars = 20 μm for (A-C), 40 μm for (D-L). (**M-P**). Quantifications of Aβ deposits (M), RPE morphological defects (N), infiltration of microglia (O), and the length of microglial dendrites. Bars depict mean ± SEM. *: P < 0.001 (*N *= 8).

Previous studies have demonstrated a link between accumulation of Aβ deposits and retinal degenerative changes in age-related macular degeneration [5,6]. Both APP and Aβ deposits have also been found in the retina of APPswe/PS1 bigenic mouse (Additional file 1, Figure 7), we therefore investigated whether CILE-induced retinal lesions are directly associated with retinal accumulation of Aβ. As shown previously [23], immunohistochemistry following labeling with a specific antibody 22C11 against for three major isoforms of human APP, reveals robust immunoreactivity in the retinal cross sections in the APPswe/PS1 bigenic mice with 6-month CILE (Additional file 1, Figure 7C), whereas only moderate APP staining and a relatively low background is found in the age-matched controls and non-Tg mice (Additional file 1, Figures 7A, B). As speculated, increased APP abundance in the retina is also followed by an increase in accumulation of Aβ deposits with CILE in the RPE as visualized by immunofluorescence microscopy following staining with 6E10 antibody (Figures 3A-F), which predominantly recognizes Aβ peptides in the current staining protocol as described [8], though 6E10 can bind to other APP fragments containing Aβ1-16 epitope. Pathological deposition of Aβ in the RPE shares very similar pattern to Aβ plaques in the brain, appearing diffusive (Figure 3C), vasculature-associated (data not shown) and/or with a condensed core (Figure 3F) as shown by confocal Z-stack microscopy. Quantification of Aβ immunoreactivity on the RPE flatmounts demonstrate a significant induction of Aβ deposits by CILE (Figure 3M).

Since CILE as well as accumulation of Aβ either intra- or extra-cellular has been linked with oxidative stress and subsequent cell damage [24], we next examined the relationship between CILE and the general architectural integrity of RPE. Accordingly, rhodamine-conjugated phalloidin was used to stain F-actin, a membrane protein that is directly relevant to the cell-cell junctions and is commonly used for assessment of junctions of cells and RPE integrity. Fluorescence microscopy demonstrates well-organized typical hexagonal shape of RPE cells in the control (Figure 3G), but disrupted staining pattern in mice that were treated with CILE (Figures 3H and 3I). Similar defect in F-actin staining is also obvious in the Aβ-immunoreactive regions (Figure 3F). Quantification of F-actin-unstained area shows a significant increase in loss of RPE integrity with CILE (Figure 3N).

### Cyclic intensive light exposure provokes neuroinflammatory response in the RPE layer in APPswe/PS1 bigenic mice

Our previous study demonstrated a link of Aβ deposition with exaggerated neuroinflammatory response in the retina of an Alzheimer's mouse [8]. As CILE significantly enhances accumulation of Aβ in the RPE layer, we further examined whether there is a corresponding change in the inflammatory process. Following immune-labeling with an antibody against IBA-1, a molecular marker for microglia, in the RPE flatmounts, fluorescence microscopy demonstrates notably increased immunoreactivity of IBA-1 in the RPE/choroid complex from the two groups of bigenic mice received CILE compared with these age-matched controls (Figures 3J-L). In addition to more cells that were stained by IBA-1, there was also a change in appearance in which IBA-1 immunoreactive cells exhibited a hypertropic appearance with much more and longer processes in the flatmounts of CILE treated mice (Figures 3J, L). Quantification of IBA-1-positive cells and total length of IBA-1-positive dendrites in examined areas indicates a significant increase in the number of microglia and the length of their dendritic processes (Figures 3O and 3P), suggesting an exacerbated inflammatory response with provoked activation of microglia in the RPE in the APPswe/PS bigenic mice after CILE.

### Upregulation of VEGF in the RPE-Choroid is related with neovascularization in the retina in APPswe/PS1 bigenic mice following cyclic intensive light exposure

**Figure 4:**
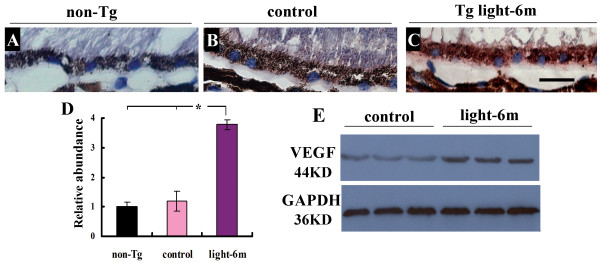
**Expression of VEGF in RPE/choroid of mice**. (**A-C**) Immunohistochemistry using a specific antibody for VEGF visualized by 3-Amino-9-ethylcarbazole (AEC, red) staining and microscopy reveals increased immunoreactivity of VEGF in the region of outer retina in mice received CILE (C) compared with both non-transgenic (non-Tg) and the bigenic control (B). Scale bar = 20 μm. (**D**) Quantification of VEGF immunoreactivity Bars depict mean ± SEM. *: P < 0.001 (*N *= 6). (**E**) Western blots using the same antibody for VEGF confirms increased immunoreactivity of VEGF in the retina of mice following 6-month CILE.

VEGF has been considered as a major player in angiogenesis and an important mediator in the pathogenesis of "wet" AMD [25,26]. To examine whether neovascularization in the outer retina induced by the CILE was on account of abnormal induction of VEGF, immunohistochemistry using a specific antibody against VEGF was performed on retinal cross sections and examined by microscopy following ABC-mediated AEC staining. When a very low basal level of VEGF signal is detected in the outer retina region from 12-month old non-Tg mice (Figure 4A), a lightly higher level of VEGF immunoreactivity is visible in the APPswe/PS1 bigenic littermates (Figure 4B). By contrast, robust staining is obvious in the 6-month CILE group (Figure 4C). Quantification of the staining results shows statistical significance of an increase in VEGF immunoreactivity in the 6-month group of CILE relative to the non-Tg as well as bigenic controls. An identical trend of VEGF expression is confirmed by Western blots (Figure 4E).

### Excessive light exposure-induced retinal lesions are associated with the functional deficits in APPswe/PS1 bigenic mice

**Figure 5:**
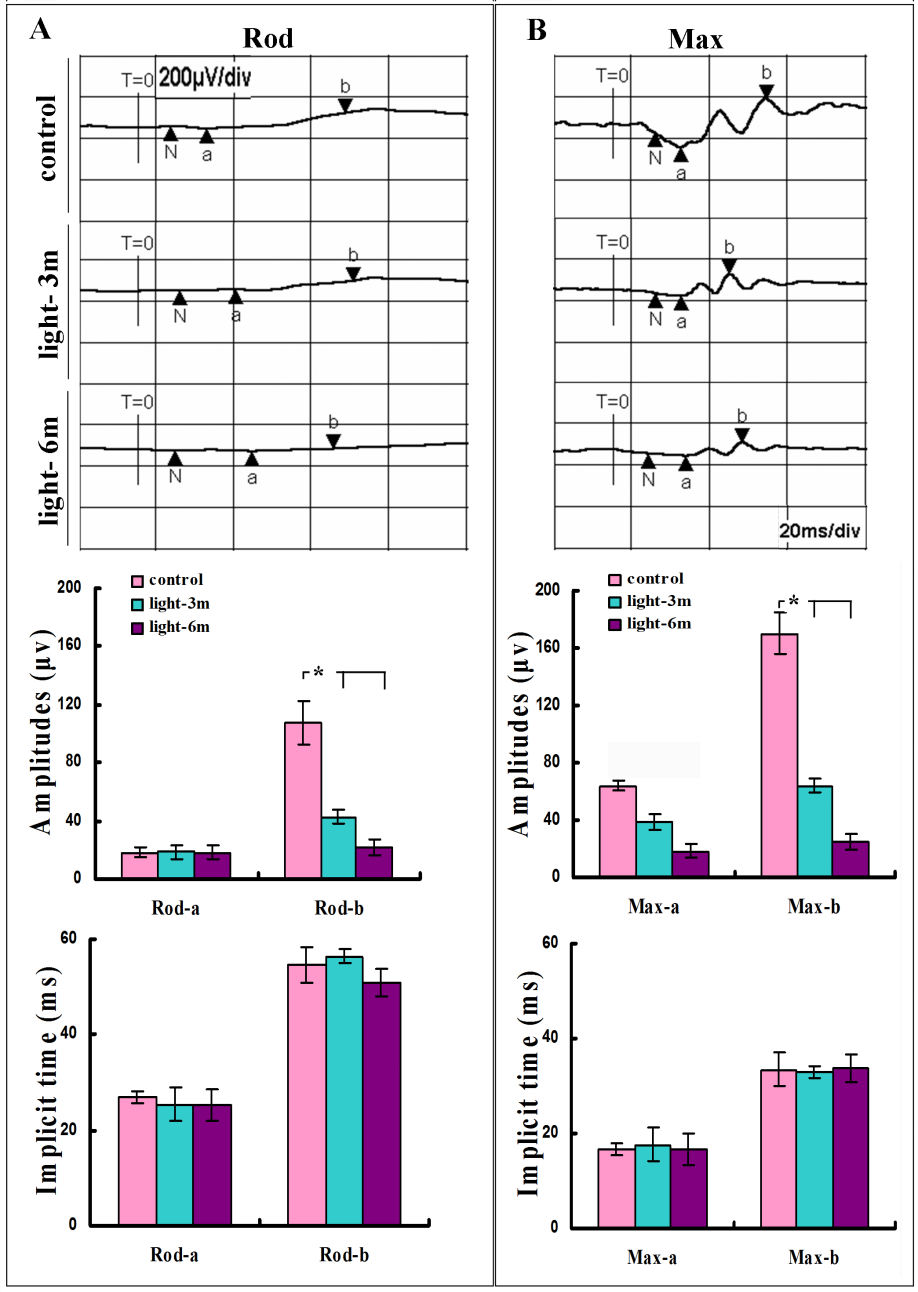
**Electroretinogram analysis in APPswe/PS1 bigenic mice**. Dark-adapted ERG amplitudes were measured in control and groups received 3-month (light-3 m) and 6-month (light-6 m) CILEs as described in the Methods. (**A**) The rod-isolated ERG response stimulated by a flash at 0.008 cd/m2 s (-25 dB). (**B**) The maximal ERG response by a flash at 2.5 cd/m^2^s (0 dB). T indicates start of the test; N, the baseline of the recording when light stimulation was given. Quantifications include measures from 12 mice for each group, i.e., *N *= 12. *: P < 0.001, Bars depict mean ± SEM.

To investigate the possibility of functional deficits as a result of CILE-induced retinal lesions, all three groups of mice were tested by scotopic ERG recording before euthanasia. Figure 5 illustrates both scotopic rod responses and maximum amplitudes after flash from three tested groups of mice. While no obvious change in *a *wave can be identified among the three groups of tested mice as the amplitudes stayed at a very low level of background, the rod response shows a conspicuous reduction in the amplitudes of *b *wave in both groups of mice received 3- or 6-month CILEs in contrast with control mice (Figure 5A), A striking decrease is also detected in the maximum amplitudes flash-triggered *b *waves in the two groups of treated mice compared with control (Figure 5B). Quantification of both scotopic rod response and the flash-triggered maximum amplitudes of *b *wave confirms the statistically significant reduction with the time of CILE. But the latency periods of *b *wave remain relatively stable in both rod and flash-induced maximum response among the three tested groups, as the implicit time stays almost unchanged in all the groups.

## Discussion

Studies have demonstrated abnormal light exposure as one of important pathogenic factors for retinopathies [15,27,28], particularly for inflammatory lesions and RPE degeneration [29,30] in the retina. Here we show that intensive light exposure exacerbates retinal degeneration with significantly exaggerated local neuroinflammatory response along with robust accumulation of Aβ deposits and CNV in the compartment of outer retina in an Alzheimer's transgenic mouse model. Although neither a typical drusen structure nor lipofuscin buildup has been identified in the model, these pathological changes are directly associated with remarkable functional deficits demonstrated by ERG recordings and well mimicked the retinopathies in AMD in human. In addition being known as a very important player in Alzheimer's disease [31,32], Aβ aggregates have been identified as a component of the drusen in AMD [6,33]. Reports from several research groups including us has evidenced deposition of Aβ aggregates in the retina in Alzheimer's transgenic mice [8,23,34], however, the data presented here are the first demonstration of not only an enhancement of Aβ aggregates by phototoxicity in the retina but also a strong link between Aβ deposition and CNV along with disrupted RPE integrity in the outer retina. Although a variety of animal models have been developed for the study of AMD, they still have their limitation to precisely recapitulate both pathological features and functional deficits of this common visual disorder. Increasing evidence has indicated an important role of Aβ in angiogenesis through induction of VEGF in Alzheimer's brain [35,36]. Interestingly, Aβ deposition was also associated with both drusenoid deposits and CNV found in the outer retina in a human apolipoprotein E4 (apoE4) knockin mouse line fed with high fat cholesterol-rich diet [9] as well as other Alzheimer's transgenic mouse lines that result in human Aβ accumulation in both brain and retina (*Tan's group, unpublished observations*). Furthermore, anti-Aβ immunotherapy protected against loss of RPE and functional performance in the apoE4 knockin mouse challenged by high fat cholesterol-rich diet [11]. Taken together, our findings further corroborate the notion about an important role of Aβ in the pathogenesis of AMD, particularly in relevance for signal transduction of CNV. In this regard, our study also suggests that an Alzheimer's transgenic mouse model with overexpression of human Aβ deposits in the retina might be a valuable animal model for AMD, when combined with other risk factors such as intensive light exposures for AMD.

Our study also revealed significant exacerbation of neuroinflammatory response in the outer retina of APPswe/PS1 bigenic mice resulting from CILE, which is in agreement with previous findings about activation of microglia in association with photoreceptor degeneration induced by intensive light exposure [37,38]. Retinal infiltration of microglia could be the crucial player to mediate the loss of photoreceptors and adjacent retinal neurons [38], since anti-inflammatory strategies can efficiently against this type of phototoxicity to the retina [39]. Importantly, Aβ has shown directly activating microglia both in vivo and in vitro [40-42]. It is therefore possible that intensive light exposure-induced retinopathies might occur through the Aβ approach. Indeed, we also detected a moderate level of APP induction in the outer retina in non-Tg mice following CILE (Figure 7, Additional file 1), though the level of Aβ is still relatively low (Figure 4A). In comparison with non-Tg mice, in fact, 6-month CILE has significantly removed photoreceptors along with adjacent outer plexiform layers (Figures 1E-J) thereby physically disabled the retinal response to light stimuli as demonstrated in ERG recording (Figure 5B). Nevertheless, retinal infiltration of microglia may be mediated through microglia-Muller cell interaction in the retina [43]. Further studies are therefore warranted to further uncover the molecular basis of light-induced Aβ accumulation and related photoreceptor degeneration.

## Conclusion

In summary, our observations demonstrate degenerative changes in the outer retina with accumulation of Aβ deposits, CNV and dramatically exaggerated neuroinflammatory response in the APPswe/PS1 bigenic mouse challenged by CILE. These results suggest that an Alzheimer's transgenic animal model with accumulation of Aβ deposits might be an alternative animal model for AMD, if combined with other confounding factors for AMD.

## Methods

### Antibodies and other reagents

Antibodies used in this study are listed in Table 1 in the Additional file 1. Tropicamide and 0.5% proparacaine were obtained from Xingqi Pharmacectical (Shengyang, China) and Alcon Laboratories (Fort Worth, Texas), respectively. The primary antibody dilution buffer, the avidin-biotin complex (ABC) kit for 3-Amino-9-ethylcarbazole (AEC) staining was bought from Boster (Wuhan, China), and 4,6-diamidino-2-phenylindole (DAPI) was purchased from Vector Laboratories (Burlingame, CA). Rodamine-conjugated phalloidin was obtained from Invitrogen (Carlsbad, CA). Total protein assay kit was from Bio-Rad (Hercules, CA). The enhanced chemiluminescence (ECL) detection system and the horse radish peroxidase-conjugated secondary antibody were purchased from Cell Signaling (Danvers, MA). Hematoxylin and eosin (H&E), and all other chemical reagents used in the experiments, unless indicated, were purchased from Sigma-Aldrich (St. Louis, MO).

### Animal and treatment

A breeding pair of the bigenic mouse line that harbors a human amyloid precursor protein with Swedish mutations (K595N/M596L, APPswe) and a mutant human presenilin 1 (PS1-dE9, PS1) were obtained from the Jackson Laboratory (Bar Harbor, ME, USA) [44]. As described, this mouse line was originally derived from a hybrid of C57BL/6 J and C3H/HeJ, which carries rd1 mutation (Pde6brd1) that confounds spontaneous retinal degeneration. To exclude possible disturbance of rd1 gene, all *rd1*-positive mice were identified by PCR genotyping and excluded in breeding (see Additional file 1). Mice were normally maintained in the institutional transgenic mouse facility with 12/12 h light-dark cycle with food and water *ad libitum*. APPswe/PS1 bigenic mice and none-transgenic littermates (non-Tg) at the age of 6 months were grouped (*N *= 12) for treatments. When the groups of control mice were kept in normal conditions, CILE for the treated groups of mice was replaced by a source of 10,000-Lux cool full spectrum light (wavelength ranges from 380 nm - 780 nm) for 3 months (light-3 m) or 6 months (light-6 m). The light intensity exposed to the animals was confirmed by a light meter (Thermo Fisher Scientific, Pittsburgh, PA). Temperature in animal cages during the light exposure was maintained between 22-24°C. To ensure the efficacy of light exposure, both eyes of each mouse were also topically given 1% atropine long-lasting emulsion once a week during the entire period of CILE. All animal procedures were conducted in accordance with the guidelines of the Association for Research in Vision and Ophthalmology Statement for the Use of Animals in Ophthalmic and Vision Research and an approval of the Institutional Animal Care and Use Committee (IACUC) of the Zhongshan Ophthalmic Center of Sun Yet-sen University.

### Fundus photography

Fundus photography was performed as described with minor modification [19]. Briefly, following papillary dilation with 1% tropicamide and 2.5% phenylephrine in HCl solution and sedation with 4.3% chloral hydrate in PBS (intraperitoneal injection, i.p.), mice were placed under the microscope operation system platform (OMS-800, Topcon, Japan) and fundus images were directly captured using a Canon G9 digital camera attached to the scope over the eye that was covered by one drop of 1% hydroxypropyl methylcellulose over the cornea to compromise refractor errors.

### Electroretinogram analysis

Scotopic electroretinogram (ERG) was recorded as reported previously [34]. Each group of animals was assayed with a RETIPORT ERG recording system (RETI Technologies, Gaithersburg, Germany). Before measuring, all animals were kept in dark overnight for at least 8 hours for dark-adaptation. Following papillary dilation with 1% tropicamide and 2.5% phenylephrine in HCl solution, sedation with 4.3% chloral hydrate in PBS (i.p.) and corneal anesthesia with 0.5% proparacaine, the gold loop electrodes were placed on the cornea, the reference electrode was plug into the mouth underneath of the tongue, and a ground electrode was subcutaneously inserted into the midway of the tail. The rod-only responses were recorded after stimulation by white light flashes with intensity of 0.008 cd/m2s (-25 dB), whereas the maximal response from the mixed rod/cone- responses (max response) was recorded with light flashes of 2.5 cd/m2s (0 dB). Three independent stimuli within 3 sec intervals were recorded as a single ERG recording. At least 3 recordings were obtained from each eye. The amplitudes and the latency of *a *and *b *waves were measured.

### Tissue preparation

Mice were euthanized with 4.3% chloral hydrate (0.1 ml/g) followed by transcardiac perfusion with ice-cold 0.1 M phosphate buffered saline (PBS, pH 7.4). Eyes were then immediately enucleated and fixed in 4% paraformaldehyde (PFA) in PBS (pH7.4) overnight at room temperature (RT) or dissected to extract retina for preparation of tissue lysates for Western blots. Fixed eyes were then preserved in PBS containing 20% sucrose and 0.05% sodium azide at 4°C, gradually dehydrated in 70% - 100% isopropanol, and embedded in paraffin blocks. Retinal cross sections (4 μm thickness) through the center of pupil-optic nerve head were prepared for histopathological analysis. To be prepared for transmission electron microscopy (EM), about 2 mm × 2 mm size of retina with attached RPE/choroid/sclera was dissected from the proximity of the optic nerve head (within 5 mm range) from some enucleated eye cups following PBS perfusion. Dissected retina-chorid-sclera tissues were immediately fixed in 2.5% glutaraldehyde and 1% PFA in 0.1 M sodium cacodylate-HCl (pH 7.4) (24 h at 4°C) followed by further post-fixation in 1% osmium tetroxide in 0.1 M cacodylate buffer-HCl (pH 7.4) for 4 h, gradually dehydrated in 50%-100% alcohol followed by immersion in propylene oxide, and embedded in epoxy resin for preparation of ultrathin sections. RPE/choroid flatmounts were prepared as described previously [45]. In general, fixed eyes were split equatorially and retinas were carefully removed from the eyecup under a stereoscope. After the extraocular muscles were removed, the posterior eye segment containing the RPE/choroid complex of each eye was spread into four quarters by four radial cuts.

### Histopathology, immunohistochemistry and immunofluorescence microscopy

Hematoxylin and eosin (H&E) staining was performed for general histopathological assessment as previously described [8]. For immunohistochemistry, paraffin-embedded retinal cross sections were deparaffinized, rehydrated, and autoclaved (121°C × 5 minutes in 10 mM citrate buffer, pH 6.0) for antigen retrieval. Following quenching of endogenous peroxidase activity with 3% H_2_O_2 _(20 min at RT), sections were blocked with 5% goat serum in PBS containing 0.1% Triton-X 100 and 20 mM L-lysine incubated with an appropriate primary antibody as listed in Table 1 (Additional file 1) at 37°C for 1 h. Specific immunoreactivity was then visualized by microscopy following incubation with a biotinylated secondary antibody, ABC kit and AEC staining. To detect Aß, outer retina flatmounts were treated by 70% formic acid for 5 min, washed with PBS, blocked with 4% bovine serum albumin in PBS containing 0.1% Triton X-100 (30 min, RT), and incubated with an appropriate primary antibody in primary antibody dilution buffer overnight at 4°C. Following appropriate washing with PBS, retinal flatmounts were incubated with an appropriate fluorophore-conjugated second antibody, counterstained with DAPI, coverslipped onto microslides, and visualized using a Zeiss LSM510 Meta Confocal Microscope. For quantification, four non-overlapped areas were randomly selected along the equatorial zone which is about 300 μm away from the optic disc, under the magnification of 400X. Images were captured using an Axioplan 2, Zeiss camera and further quantified with Image Pro-Plus1.42q (National Institutes of Health, USA). Total Aß-positive area, number of IBA-1-immunoractive cells, and total length of IBA-1-immunoractive dendrites of microglia within the four selected regions were quantified. Disorganization of RPE alignment was scored as percentage of phalloidin-negative area over the entire RPE flat mounts.

### Western blot

Western blotting was conducted as previously described [46,47]. Briefly, dissected retinas were homogenized in lysis buffer (50 mM Tris-HCl/pH7.5, 5 mM EDTA, 150 mM NaCl, 0.5% NP-40, protease inhibitor cocktail mixture) and pelleted. The concentrations of total protein in the supernatants were assayed using Bio-Rad Protein Assay kit. About 35 μg total protein of lysate from each samples was resolved in 10% SDS-polyacrylamide gel, blotted on PVDF membrane followed by incubation with Blotto (0.1% Tween-20 and 5% milk in PBS, × 1 h), a specific primary antibody in 5% milk in PBST (overnight at 4°C) and with a horse radish peroxidase-conjugated secondary antibody in RT with appropriate washing. The immunoreactivity was visualized with ECL.

### Transmission electron microscopy

The ultrathin (70 nm) sections were prepared using a Leica UltraCut S Microtome, counterstained with uranyl acetate and lead citrate, and examined under a JEOL 100CX II electron microscope (JEOL, Tokyo, Japan) at 80 kV. Microscopic images were acquired using X-ray films.

### Statistical analysis

In all of the graphs, the data points represent the means ± S.E.M. from all individuals in each group of animals (*N *= 6-12). Applicable comparisons were performed by one-way analysis of variance followed by Student's *t*-test for multiple groups or independent samples or *T*-test for two groups by SPSS13.0 software. The difference between groups was considered as statistically significant when the value of p was ≤ 0.05.

## Abbreviations

APP: Amyloid precursor protein; Aβ: Beta-amyloid; AMD: Age-related macular degeneration; APPswe: Human amyloid precursor protein with Swedish mutations; BrM: Bruch's membrane; AEC: 3-Amino-9-ethylcarbazole; CILE: Cyclic intensive light exposure; CNV: Choroidal neovascularization; CC: Choroicapillaris; DAPI: 4,6-diamidino-2-phenylindole; ERG: Electroretinogram; ECL: Enhanced chemiluminescence; EM: Transmission electron microscopy; H&E: Hematoxylin and eosin; IBA-1: Ionized calcium binding adaptor molecule-1; IACUC: The Institutional Animal Care and Use Committee; INL: Inner nuclear layer; IPL: Inner plexiform layer; non-Tg: None transgenic littermates; OPL: Outer plexiform layer; ONL: Out nuclear layer; OS: Photoreceptor outer segment; OCL: Outer collagenous layer; PS1: Presenilin 1; PCR: Polymerase chain reaction; PFA: Paraformaldehyde; PBS: Phosphate buffered saline; RT: Room temperature; RGC: Retinal ganglion cell; RPE: Retinal pigment epithelium; VEGF: Vascular endothelial growth factor.

## Competing interests

The authors declare that they have no competing interests.

## Authors' contributions

ZD and JL conceived of the study and experimental design and drafted manuscript. ZD and JL carried out experiments. ZD, JL, YL, XL, HH and YH helped experimental design and data analysis. ZT initiated original idea for the study and steered data presentation and manuscript drafting and revision. JG funded the study, guided conceiving of the study and experiments, and helped data analysis, manuscript drafting and revision. All authors read and approved the final manuscript.

## Supplementary Material

Additional file 1 **Supplementary data **[48-54]. Click here for file
