# Supplementary material for: Cyclic intensive light exposure induces retinal lesions similar to age-related macular degeneration in APPswe/PS1 bigenic mice
Source: BMC Neurosci. 2012 Mar 24;13:34. doi: 10.1186/1471-2202-13-34 (PMC3338397; doi:10.1186/1471-2202-13-34)
Supplement: Additional file 1 — Supplementary data [48-54]. [file 1471-2202-13-34-S1.PDF]

Supplementary data

Table 1. List of antibodies used in the study

Name and related antigen	Tg type	Source	Dilution	References
22C11 for human APP	Mouse monoclonal IgG	Chemicon (Bellerica, MA)	1/100	[1]
6E10 for Aβ	Mouse monoclonal IgG	Covance (Denver, PA)	1/100	[2]
anti-GAPDH	Mouse monoclonal IgG	Kangcheng (Shanghai, China)	1/500	
CP290A for IBA1	Rabbit polyclonal IgG	Biocare (Concord, CA)	1/200	[3]
anti-VEGF	Rat polyclonal IgG	Beyotime (Wuhan, China)	1:500	[4]
Cy3-conjugated goat anti-rat IgG	Goat polyclonal IgG	Boster (Wuhan,China)	1/100	--
FITC-conjugated goat anti-rabbit IgG	Goat polyclonal IgG	Boster (Wuhan,China)	1/100	--
Cy3-conjugated goat anti-rabbit IgG	Goat polyclonal IgG	Boster (Wuhan,China)	1/100	--
FITC-conjugated goat anti-mouse IgG	Goat polyclonal IgG	Boster (Wuhan,China)	1/100	--
Cy3-conjugated donkey anti-mouse IgG	Donkey polyclonal IgG	Boster (Wuhan,China)	1/100	--

PCR Genotyping

PCR genotyping was conducted as described [5, 6]. Briefly, genomic DNA was extracted from about 0.5 cm tail tips [7]. Primers used for detection of *rd1*/Pde6b^{rd1} gene are: 5'-ATGTACCGCCAGCGCAATGG-3' (forward) and 5'-CCCCGCCTTCTCAACAACCTGGGA CGGGAG-3' (reverse) [8]. PCR reaction included: 94°C x 3 min and,94°C x 0.5 mine, 60°C x 0.5 min, and 72°C x 1 min for 40 cycles followed by 72°C x 2 minute extension and stopped at 10 °C.

Figures

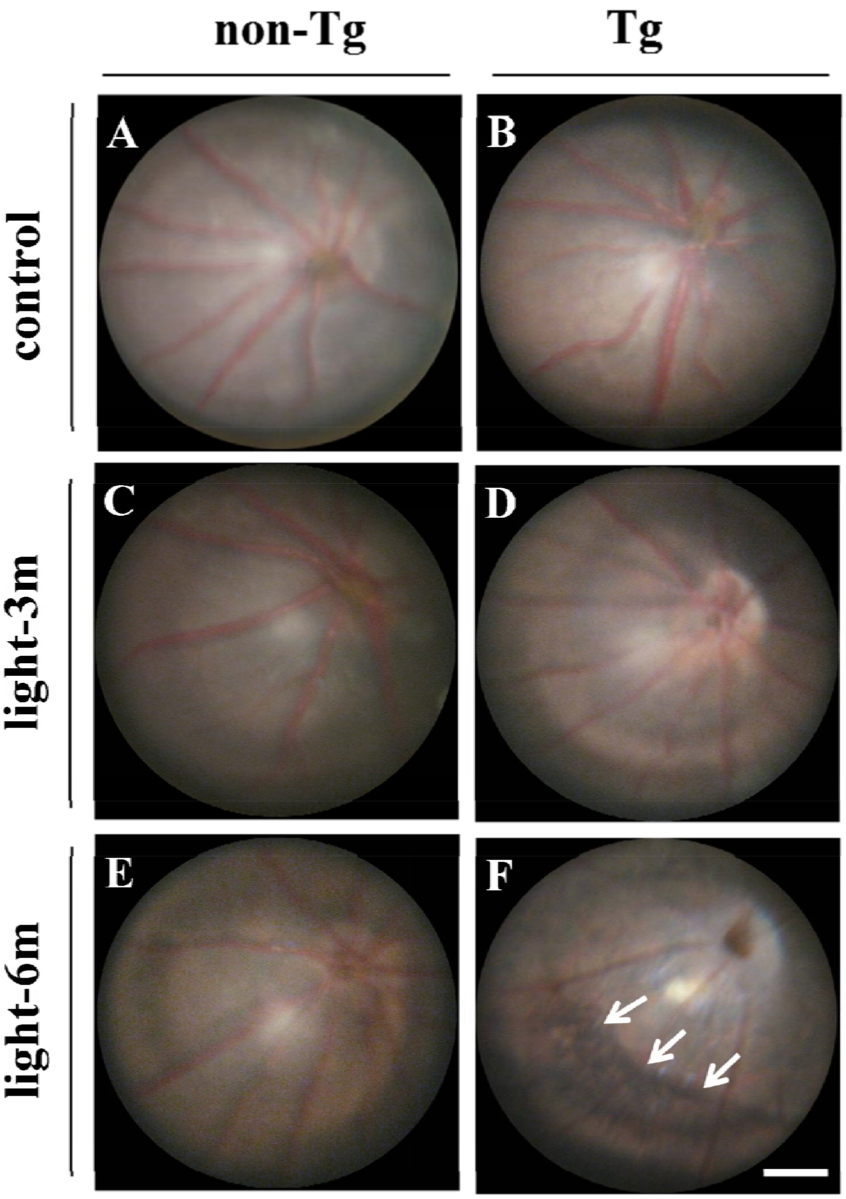

Figure 6. Fundus photographs of eyes. Resembling normal appearance of the retinal fundus was detected in 12-month old *rd1*-negative non-transgenic (non-Tg, **A**) and APP/PS1 bigenic (Tg, **B**) mouse eyes. Three or six months after enhanced lighting exposures no significant changes were found in the non-Tg mouse eyes (**C** and **E**), whereas Tg mouse eyes (**D** and **F**) demonstrated shrunken vessels and increased pigment deposits (arrows). Scale bar = 200μm.

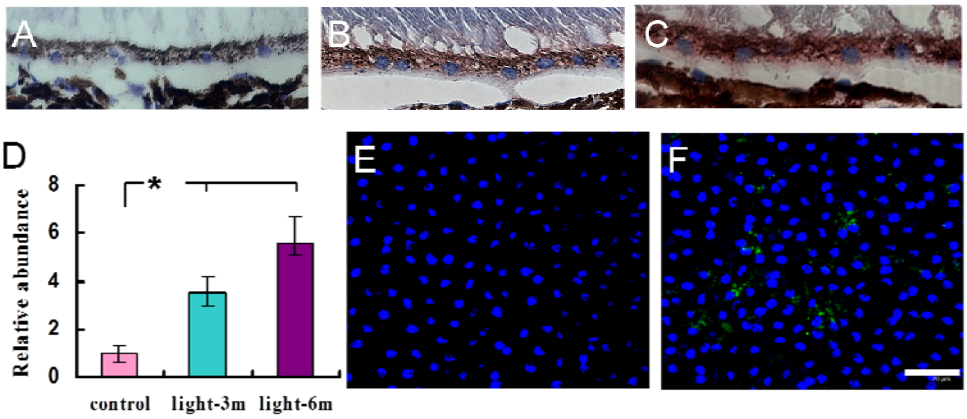

Figure 7. Abundance of APP and Aβ deposits in the RPE layer of APP/PS1 bigenic (Tg) mice and non-transgenic littermates (non-Tg) assayed by immunohistochemistry. (**A-C**) Immunohistochemistry using 22C11 specific antibody for APP visualized by AEC (dark red) staining and microscopy reveals increased immunoreactivity of APP in the region of outer retina in Tg control and Tg mice received 6-month excessive light exposures (**C**) compared with both non-transgenic (non-Tg) and the bigenic control (**B**). Immunofluorescence following 6E10 antibody for Aβ demonstrates low background in the RPE flatmount in non-Tg control (**E**) and robust staining (green) in 12-month old APP/PS1 bigenic mice (**F**). Cell nuclei are counterstained blue by 4'-6-diamidino-2-phenylindole (DAPI). Scale bar = 40μm. (**D**) Quantification of APP immunoreactivity P <0.001(**F**). *: P <0.001, Error bars depict mean ± SEM.

References:

1. Martin LJ, Sisodia SS, Koo EH, Cork LC, Dellovade TL, Weidemann A, Beyreuther K, Masters C, Price DL: **Amyloid precursor protein in aged nonhuman primates**. *Proc Natl Acad Sci U S A* 1991, **88**(4):1461-1465.

2. Thakker DR, Weatherspoon MR, Harrison J, Keene TE, Lane DS, Kaemmerer WF, Stewart GR, Shafer LL: **Intracerebroventricular amyloid-beta antibodies reduce cerebral amyloid angiopathy and associated micro-hemorrhages in aged Tg2576 mice**. *Proc Natl Acad Sci*

U S A 2009, **106**(11):4501-4506.

3. Ito D, Imai Y, Ohsawa K, Nakajima K, Fukuuchi Y, Kohsaka S: **Microglia-specific localisation of a novel calcium binding protein, Iba1**. *Brain Res Mol Brain Res* 1998, **57**(1):1-9.

4. Wakisaka Y, Chu Y, Miller JD, Rosenberg GA, Heistad DD: **Spontaneous intracerebral hemorrhage during acute and chronic hypertension in mice**. *J Cereb Blood Flow Metab*, **30**(1):56-69.

5. Zhang B, Veasey SC, Wood MA, Leng LZ, Kaminski C, Leight S, Abel T, Lee VM, Trojanowski JQ: **Impaired rapid eye movement sleep in the Tg2576 APP murine model of Alzheimer's disease with injury to pedunculopontine cholinergic neurons**. *Am J Pathol* 2005, **167**(5):1361-1369.

6. Pratico D, Uryu K, Leight S, Trojanoswki JQ, Lee VM: **Increased lipid peroxidation precedes amyloid plaque formation in an animal model of Alzheimer amyloidosis**. *J Neurosci* 2001, **21**(12):4183-4187.

7. Jankowsky JL, Slunt HH, Ratovitski T, Jenkins NA, Copeland NG, Borchelt DR: **Co-expression of multiple transgenes in mouse CNS: a comparison of strategies**. *Biomol Eng* 2001, **17**(6):157-165.

8. Bowes C, Li T, Frankel WN, Danciger M, Coffin JM, Applebury ML, Farber DB: **Localization of a retroviral element within the rd gene coding for the beta subunit of cGMP phosphodiesterase**. *Proc Natl Acad Sci U S A* 1993, **90**(7):2955-2959.
